# Reactive oxygen species from mitochondrial complex III are induced by NCLX to promote astrocytic STAT3‐linked transcription and dementia‐related pathology

**DOI:** 10.1002/alz.084464

**Published:** 2025-01-03

**Authors:** Daniel Barnett, Till S Zimmer, Caroline Booraem, Fernando Palaguachi, Samantha M. Meadows, Haopeng Xiao, Edward T Chouchani, Anna G. Orr, Adam L Orr

**Affiliations:** ^1^ Appel Alzheimer’s Disease Research Institute, Weill Cornell Medicine, New York, NY USA; ^2^ Neuroscience Graduate Program, Weill Cornell Medicine, New York, NY USA; ^3^ Weill Cornell Medicine, New York, NY USA; ^4^ Feil Family Brain and Mind Research Institute, Weill Cornell Medicine, New York, NY USA; ^5^ Weill Cornell Medicine, New York, NY, NY USA; ^6^ Department of Cancer Biology, Dana‐Farber Cancer Institute, Boston, MA USA; ^7^ Department of Cancer Biology, Harvard Medical School, Boston, MA USA; ^8^ Weill Cornell Medicine‐Rockefeller‐Sloan Kettering Tri‐Institutional MD‐PhD Program, New York, NY USA

## Abstract

**Background:**

Mitochondrial reactive oxygen species (mROS), such as superoxide and hydrogen peroxide (H_2_O_2_), are implicated in aging‐associated neurological disorders, including Alzheimer’s Disease and frontotemporal dementia. Mitochondrial complex III of the respiratory chain has the highest capacity for mROS production and generates mROS toward the cytosol, poising it to regulate intracellular signaling and disease mechanisms. However, the exact triggers of complex III‐derived ROS (CIII‐ROS), its downstream molecular targets, and its functional roles in dementia‐related pathogenesis remain unclear.

**Method:**

Here, we investigated the drivers and consequences of CIII‐ROS production in primary mouse astrocytes and mouse models of dementia‐linked proteinopathy using site‐selective mROS suppressors and genetic approaches together with live‐cell imaging of sub‐compartmental H_2_O_2_ dynamics, stoichiometric redox proteomics, and transcriptomics.

**Result:**

We found that specific disease‐related factors transiently increase astrocytic CIII‐ROS levels, and this effect is dependent on a mitochondrial sodium‐calcium exchanger. CIII‐ROS oxidized specific cysteines on astrocytic proteins associated with disease, amplified STAT3 phosphorylation and nuclear translocation, and promoted gene expression changes linked to STAT3 and related neuroimmune pathways. Inhibition of other sites of ROS production, including mitochondrial complex I and NADPH oxidase, had no effects on mROS responses or STAT3 signaling, demonstrating the specificity of CIII‐ROS induction and its context‐dependent modulation of STAT3 activities. Blockade of CIII‐ROS in transgenic mouse models of dementia with a site‐selective and brain‐penetrant suppressor reduced astrocytic alterations, neuropathology, and premature mortality.

**Conclusion:**

Our data suggest that dementia‐associated factors induce site‐specific mROS production and that CIII‐ROS promote precise post‐translational redox modifications and amplify STAT3‐linked signaling and changes in gene expression. Together, our findings reveal CIII‐ROS as an important node of mitochondrial‐nuclear communication in pathogenic conditions, whereby mROS transients are converted into long‐lasting changes in gene transcription and cell function. CIII‐ROS and redox signaling offer new therapeutic opportunities for aging‐associated neurological disorders.

